# P-399. Antibiotic Prescribing in Pediatric Patients with Neurodevelopmental Disorders in Ambulatory Settings

**DOI:** 10.1093/ofid/ofaf695.616

**Published:** 2026-01-11

**Authors:** Anh N Vo, Prachi Singh, Megumi Okumura

**Affiliations:** UCSF Benioff Children's Hospitals Oakland & San Francisco, San Francisco, CA; UCSF Benioff Children's Hospital Oakland, Oakland, California; UCSF Benioff Children's Hospitals San Francisco, San Francisco, California

## Abstract

**Background:**

Upper respiratory infections and acute otitis media are the most common reasons for pediatric ambulatory visits. Though mostly viral and self-resolving, antibiotics are often prescribed. Neurodevelopmental disorders (NDDs) include intellectual developmental disorders, communication disorders, autism spectrum disorder, attention-deficit/hyperactivity disorder, specific learning disorder, and motor disorders. Children with NDD face health disparities, but little is known about antibiotic prescribing patterns for this group.

**Methods:**

We conducted a retrospective cross-sectional study using de-identified electronic health record data. Our inclusion criteria included the following:

- Pediatric patients (age ≤18 years) with ambulatory encounters between 1/1/2020 and 12/31/24 with a diagnosis of a URI and AOM, identified using ICD-10 codes

Our exclusion criteria included the following:

- Patients with history of cancer (e.g., leukemia, CNS tumors, lymphoma, and other childhood cancers)
- Encounters with concurrent diagnoses urinary tract infection, pyelonephritis, and skin soft tissue infection

Patients classified as having NDD if they had at least two encounters with the same NDD ICD-10 code prior to the URI encounter. All analyses were performed using R. Chi-square testing and logistic regression were used.

**Results:**

URI: Encounters for patients with NDD had antibiotics for URIs at significantly higher rates (p=0.0154). However, when controlling for age, department, URI category, and race/ethnicity in logistic regression, NDD status alone did not significantly predict antibiotic prescribing (p=0.4949).

AOM: Encounters for patients without NDD were prescribed more antibiotics, but this result was not statistically significant (p-value of *0.4242*).

**Conclusion:**

This is the first study to our knowledge examining antibiotic prescribing practices for children with neurodevelopmental disorders. Our analysis for URI and AOM revealed mixed results, though the results were not statistically significant. Further research can help understand whether differences in antibiotic prescribing exist for other common pediatric conditions, in the inpatient setting, and regarding choice and duration of therapy for children with NDD vs children without NDD.

**Disclosures:**

All Authors: No reported disclosures

